# Biogas residue parameterization for soil organic matter modeling

**DOI:** 10.1371/journal.pone.0204121

**Published:** 2018-10-12

**Authors:** Nadia Prays, Peter Dominik, Anja Sänger, Uwe Franko

**Affiliations:** 1 Department of Bioenergy and Department of Soil System Science, Helmholtz Centre for Environmental Research–UFZ, Halle, Germany; 2 Department of Ecology, Chair of Soil Science, Technische Universität Berlin, Berlin, Germany; 3 Department of Technology Assessment and Substance Cycles, Leibniz Institute for Agricultural Engineering Potsdam-Bornim, Potsdam, Germany; 4 Department of Soil System Science, Helmholtz Centre for Environmental Research–UFZ, Halle, Germany; Oak Ridge National Laboratory, UNITED STATES

## Abstract

A variety of biogas residues (BGRs) have been used as organic fertilizer in agriculture. The use of these residues affects the storage of soil organic matter (SOM). In most cases, SOM changes can only be determined in long-term observations. Therefore, predictive modeling can be an efficient alternative, provided that the parameters required by the model are known for the considered BGRs. This study was conducted as a first approach to estimating the organic matter (OM) turnover parameters of BGRs for process modeling. We used carbon mineralization data from six BGRs from an incubation experiment, representing a range of substrate inputs, to calculate a turnover coefficient k controlling the velocity of fresh organic matter (FOM) decay and a synthesis coefficient η describing the SOM creation from FOM. An SOM turnover model was applied in inverse mode to identify both parameters. In a second step, we related the parameters k and η to chemical properties of the corresponding BGRs using a linear regression model and applied them to a long-term scenario simulation. According to the results of the incubation experiment, the k values ranged between 0.28 and 0.58 d^-1^ depending on the chemical composition of the FOM. The estimated η values ranged between 0.8 and 0.89. The best linear relationship of k was found to occur with pH (R^2^ = 0.863). Parameter η is related to the C_t_/N_org_ ratio (R^2^ = 0.696). Long-term scenario simulations emphasized the necessity of specific k and η values related to the chemical properties for each BGR. However, further research is needed to validate and improve these preliminary results.

## 1 Introduction

In recent decades, there has been increasing interest in Europe in the use of anaerobic digestion plants in farming contexts because of EU policies and directives aimed at reducing greenhouse gases (GHG) and the promotion of renewable energy production [1]. The expansion of biogas production in Germany began with the adoption of the Renewable Energy Sources Act in 2000 and its amendment in 2004. Germany’s current policy strives to increase the contribution of renewable energy resources as a substitute for fossil energy resources in order to decrease CO_2_ emissions [2]. Thus, biogas production will be a key technology in the future.

By 2015 in Germany, approximately 8,000 biogas plants were producing biogas from different biomass sources [3]. During the production of biogas, an organic byproduct, known as BGR, is produced in large quantities. BGRs contain high levels of plant-available nutrients (N, P, K) and a considerable amount of organic carbon, which explains their widespread usage in agriculture for closing nutrient cycles [4, 5]. Several studies have demonstrated the benefits of the agricultural use of BGRs for yields and soil fertility [6, 7]. Anaerobic digestion may transform 20–95% of the carbon in the feedstock into gaseous carbon compounds. Consequently, the amount of organic carbon introduced into the soil is reduced in comparison with direct soil incorporation of undigested organic residues [8]. Furthermore, anaerobic digestion increases the availability of N (NH_4_-N) due to the breakdown of organically bound N during the anaerobic process [9, 10]. Odlare, Pell [11] found that, relative to other treatments (pig manure, cattle manure, compost, inorganic fertilizer), soils treated with BGRs from household wastes displayed the highest microbial biomass, nitrogen mineralization rate and potential ammonia oxidation. Thus, BGRs may have an effect on the storage of soil organic matter (SOM) and the nitrogen balance in the soil, both of which are important for sustainable soil use and the maintenance of soil functions. Since the impact of BGRs on SOM changes can only be determined in long-term applications, predictive modeling can be a helpful tool to estimate changes in SOM. Several process-based agroecosystem models, such as DNDC and RothC, have been widely utilized for quantifying soil carbon sequestration and are capable of determining the effects of organic fertilizers on soil carbon dynamics [12]; however, until now, no model considers BGRs. It is crucial to define the carbon turnover parameters of BGR degradability and their efficiency for creating new SOM when modeling SOM dynamics [13, 14].

Some of the required parameters, such as dry matter and carbon concentration, are fast and easy to obtain for modeling. However, to derive the carbon turnover parameters, incubation experiments over a period of several weeks are usually required. [14]. Incubation experiments are time consuming, and in practical applications, it is not possible to perform incubation experiments regularly. Furthermore, since the substrate mix applied for biogas production is very heterogeneous in terms of the composition of plant nutrients and organic matter, the chemical composition of BGRs is also very diverse [10, 15]. Therefore, a specific parameter set may be required for each BGR.

The objective of this study was to derive BGR carbon turnover parameters for process modeling. Therefore, we first used the carbon mineralization rates from an incubation experiment, which is described in detail in Sänger, Geisseler [16], to calculate the parameters for BGRs with different composition. In a second step, we hypothesized that it is possible to estimate these parameters using some easily measurable BGR properties and to develop a simpler approach as an alternative to the existing time- and labor-consuming incubation experiments.

## 2 Material and methods

### 2.1 Biogas residues

We chose only BGRs that had been sampled from an additional BGR store and not directly from the fermenter. This was done to ensure a comparable (high) degree of degradation of all BGRs for our parameterization. The BGRs were not chosen on the basis of the substrate input combination. The BGRs used in this study represent a wide range of substrate inputs (Table 1) and were taken from two-stage biogas plants. The indices refer to the percentage of maize in the substrate input. Other compounds include grass silage, rye silage, cereals, pig slurry, cattle slurry and farmyard manure. All of the BGRs were derived from wet digestion under mesophilic conditions. The methods used for BGR analysis are described in detail in Sänger, Geisseler [16]. The chemical properties of used BGRs are shown in Table 2.

**Table 1:** Composition of BGRs (in % mass) from Sänger, Geisseler [16].

BGR	maize silage	grass silage	rye silage (whole plant)	shredded grain	pig slurry	cattle slurry	farmyard manure
	%	%	%	%	%	%	%
D_17_	17	-	-	-	19	64	-
D_24_	24	31	8	-	-	37	-
D_33_	33	-	25	-	20	-	22
D_52_	52	8	-	2	35	-	3
D_61_	61	-	-	5	34	-	-
D_100_	100	-	-	-	-	-	-

**Table 2:** Chemical properties of BGRs from Sänger, Geisseler [16]. DM = dry matter, DM_org_ = organic dry matter, C_t_ = total carbon, N_t_ = total Kjeldahl nitrogen, N_org_ = organic nitrogen calculated as N_org_ = N_t_-NH_4_-N.

BGR	DM	DM_org_	pH	C_t_	NH_4_-N	N_org_	N_t_	C_t_/N_t_	C_t_/N_org_
	%	%		%DM	%DM	%DM	%DM		
D17	5.5	28.1	8	38.4	2.9	3.4	6.3	6.1	11.3
D24	9.2	27.9	7.8	39.2	3.5	3.0	6.5	6.0	13.1
D33	9.6	33.0	8	41.3	2	3.0	5.0	8.3	13.8
D52	7.2	29.2	7.7	40.7	4.3	3.4	7.7	5.3	12.0
D61	8.1	33.48	7.9	42	5.5	2.7	8.2	5.1	15.6
D100	6.8	34.08	7.7	43.2	2.9	4.3	7.2	6.0	10.0

### 2.2 Model and carbon turnover parameter description

We used the carbon turnover sub-model that is integrated in the agroecosystem model CANDY (Carbon And Nitrogen DYnamics) and the SOM model CCB (Candy Carbon Balance) [17, 18]. Here, SOM is divided into an active pool where mineralization occurs, a stabilized pool that represents the passive but decomposable part of the SOM, and a long-term stabilized pool that is considered inert. Matter exchange between the active SOM and stabilized SOM is assumed by the model. A more detailed description of the interaction between these model pools was given by Franko, Kolbe [18]. In our model, the carbon reproduction (C_rep_) flux, representing that part of FOM that is incorporated within the active SOM pool, is the driver of SOM accumulation. The FOM turnover is described using first-order kinetics with the following parameters: 1) a turnover coefficient k describing the resistivity against microbial breakdown of the material and 2) a synthesis coefficient η specifying the carbon transfer from decomposed FOM to new active SOM. Thus, higher k values indicate a higher velocity of FOM decay. Higher η values indicate higher contributions to SOM.

Furthermore, the BGRs need to be characterized based on the following properties: organic carbon, organic and inorganic nitrogen, total dry matter for quantitative modeling of matter fluxes. All values should be given as concentration of % weight by weight (% w/w).

### 2.3 Incubation

The incubation experiment including the CO_2_ emission data is described in detail in Sänger, Geisseler [16] and is briefly summarized here. Two different soils were used for the incubation experiment: a silty soil (5% sand, 75% silt, 20% clay, pH value = 6.5) and a sandy soil (46% sand, 39% silt, 15% clay, pH value = 7.5). The dried and sieved soils were amended with each of the BGRs at a rate of 0.5 g N (kg soil)^−1^, adjusted to 60% of water holding capacity, and incubated at 25°C for 6 weeks. Emissions of CO_2_ were measured daily on days 1–20, afterwards on days 22, 24, 27, 30, 34, 36, 41. The mineralization rates were calculated as part of the added carbon. All of the BGRs and control treatments were incubated in four replications. The observed mineralization rates were cumulated in the following modeling step. The control treatments were measured with another frequency; thus, the missing values were interpolated to a polynomial. Differences between each sample and each control treatment were calculated for each observation time step. These differences were used to calculate a mean cumulative mineralization rate value and the total variance of each BGR and time step. For our analysis, we selected only the first 20 days of the experiment for daily measurements.

### 2.4 Inverse modeling

We used inverse modeling to fit the parameters k and η to the observed CO_2_ mineralization for each BGR–soil combination, minimizing the root mean square error (RMSE) between the observed and modeled values. The fitting procedure is based on a numerical simplex method for minimization of a non-linear function [19]. The parameter estimation was completed using uncertainty calculations based on the Fisher Information Matrix approach [20]. This approach is based on the sensitivity of the model output and the variance of the measured CO_2_ emissions at each observation point. For further analysis, we took the mean values of k and η for each BGR because, according to the model approach, k and η depend only on FOM type. Site conditions, such as soil type, are considered in the model using the Biological Active Time (BAT), which was calculated for each soil separately [21].

### 2.5 Prediction of model parameters based on chemical properties

In the next step, the estimated carbon turnover parameters from inverse modeling were related to the chemical properties of BGRs. This was done to predict the model parameters without using time-consuming incubation experiments. Therefore, a linear regression (*f(x) = mx+n*) was performed and the coefficient of determination R^2^ was calculated; the slope *m* and intercept *n* were calculated using R version 3.3.1 (The R Foundation for Statistical Computing, 2016). For the x variable, the chemical properties dry matter (DM), organic dry matter (DM_org)_, total carbon (C_t_), total Kjeldahl nitrogen (N_t_), ammonium nitrogen (NH_4_-N), organic nitrogen calculated as N_org_ = N_t_-NH_4_-N, C_t_/N_t_, C_t_/N_org_ and pH were used.

The approach with the best R^2^ was selected. In the following text, we use the symbols k and η to represent the parameters that were estimated with inverse modeling and k* and η* to represent when their predictions were based on the chemical properties of the BGRs.

### 2.6 Scenario modeling

A simple bioenergy production scenario of continuous maize (yield = 500 dt ha ^-1^) and annual BGR application as organic fertilizer (170 kg N ha ^-1^) was modeled with the CCB model that was already validated [18]. The influence on carbon storage for each BGR was evaluated using the mean, minimal and maximal k and η as well as k* and η*. The influence on carbon storage was evaluated using k and η as well as k* and η* for each BGR. The parameter errors were included in the simulation; thus, for each parameter the mean, minimal and maximal values were calculated. For the scenario simulation, we assumed typical site conditions of the Chernozem region in Central Germany, with a mean annual temperature of 8.5°C, mean annual precipitation of 480 mm and Haplic Chernozem soil (21% clay, 68% silt, 11% sand). The initial value for soil organic carbon was set to 2%. Soil organic carbon change was calculated for 100 years of unchanged soil management. The mean C_org_ concentrations as well as the standard deviations were calculated. The effects of different treatments were analyzed with a one-way ANOVA. A least significant difference t-test (LSD) was used to compare the mean values and to assess the significance of the differences between the mean values. The effects were considered significant for p < 0.05. All statistical analyses were performed using R version 3.3.1 (The R Foundation for Statistical Computing, 2016).

## 3 Results

### 3.1 Estimation of parameters from incubation results

**Fig 1:**
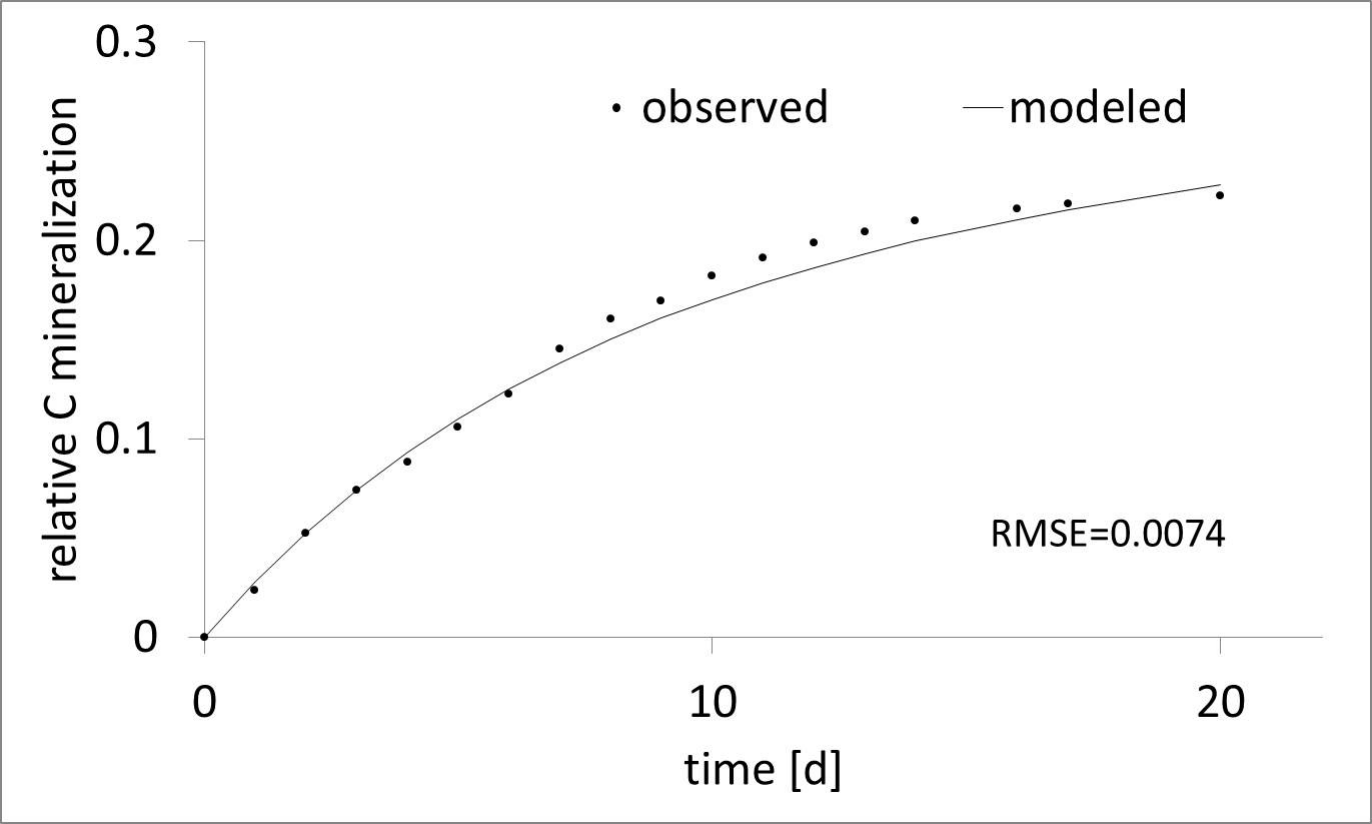
Measured and modeled relative C mineralization of BGR with a biggest RMSE (D_100_).

Using inverse modeling to identify the parameters from the observed CO_2_ emissions provided good results (Fig 1). The adaptation error was lower than 1% of emitted carbon for all treatments (Table 3). Our estimated k values were between 0.28 and 0.58 d^-1^, and the η values of BGRs were between 0.8 and 0.89 (Table 3).

**Table 3:** Fitted parameterized values of six different BGRs. RMSE = root mean square error between the modeled and observed values of C mineralization, sd standard deviation of the fitted parameters (see also S1 Table).

BGR	k		Η		RMSE
	[d^-1^]	sd	[–]	sd	[part of emitted C]
D_17_	0.362	0.060	0.844	0.009	0.007
D_24_	0.420	0.075	0.871	0.018	0.008
D_33_	0.279	0.036	0.828	0.018	0.006
D_52_	0.506	0.081	0.851	0.016	0.008
D_61_	0.391	0.034	0.802	0.015	0.009
D_100_	0.575	0.076	0.890	0.009	0.009

### 3.2 Prediction of model parameters based on chemical BGR properties

**Fig 2:**
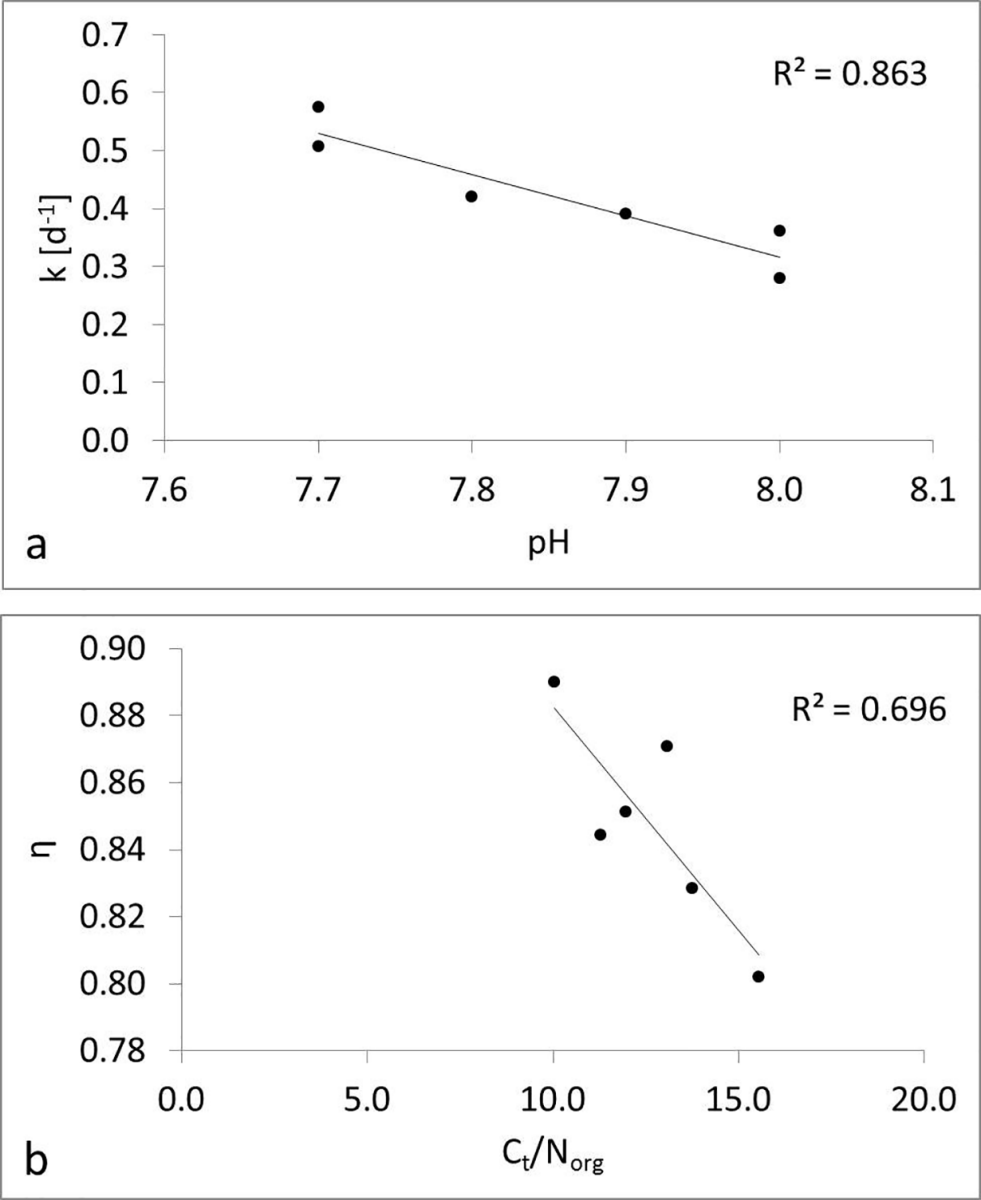
a) relationship and R^2^ of k and pH, b) relationship and R^2^ of η and C_t_/N_org_.

The results of the regression analysis are shown in Table 4. Parameter k in d^-1^ was strongly related to the pH values: k = 5.996–0.710*pH (R^2^ = 0.863) (Fig 2A). Synthesis coefficient η was best described using C_t_/N_org_ ratio: η = 1.016–0.013* C_t_/N_org_ (R^2^ = 0.696) (Fig 2B). Multiple regressions were not considered because the correlations between C/N_org_ and the pH value (r = 0.43), and N_org_ and the pH value (r = 0.55) were too high. The correlation between the η and the C/N_org_ ratio, k and pH value were found to be significant (p<0.05).

**Table 4:** R^2^ of the linear relationship between the model parameters k or η and selected the BGR property.

BGR property	R^2^ (k)	R^2^ (η)
DM	0.245	0.046
DM_org_	0.035	0.139
C_t_	0.180	0.001
NH_4_-N	0.064	0.189
N_org_	0.547	0.624
N_t_	0.402	0.007
C_t_/N_t_	0.495	0.028
C_t_/N_org_	0.371	0.696
pH	0.863	0.411

### 3.3 Modeled vs. predicted parameters–a comparison of results

**Fig 3:**
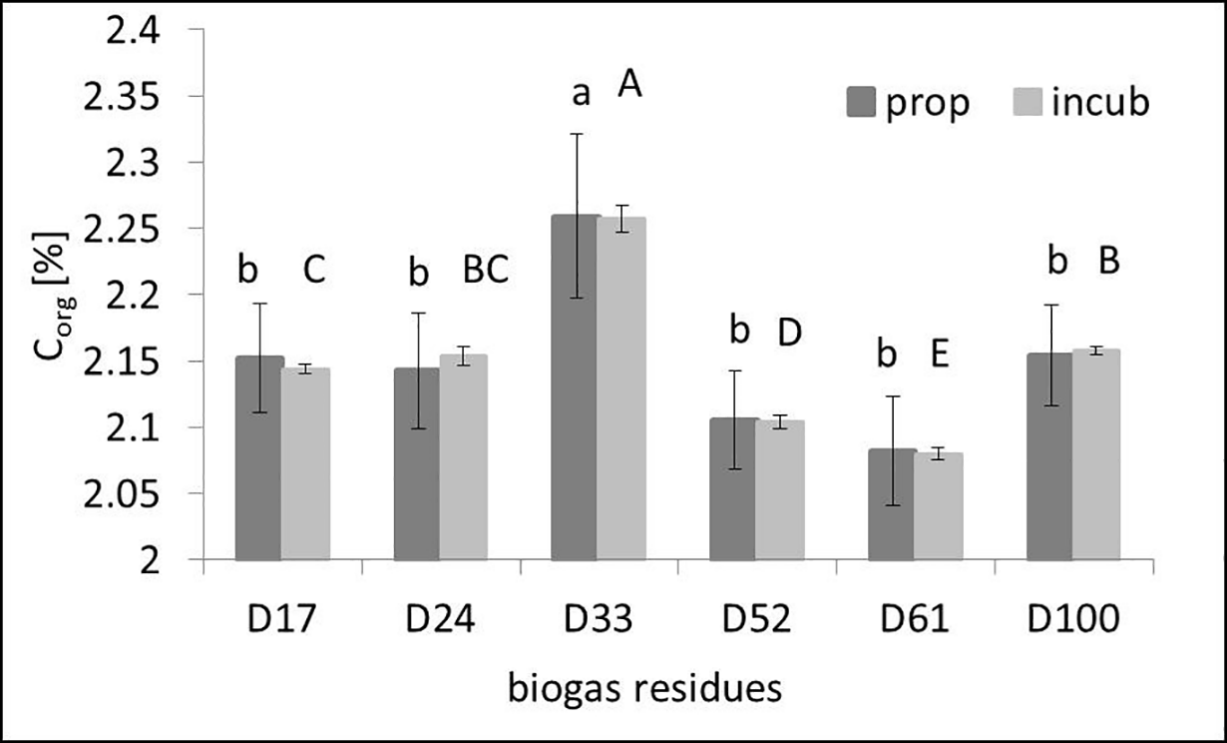
C_org_ concentrations after a 100-year scenario simulation with continuous maize (yield 500 dt ha ^-1^) and BGR application (170 kg N ha ^-1^). incub = parameters estimated with the results of the incubation experiment (k, η), which were used for the modeling; prop = parameters predicted with chemical properties of the BGRs (k*, η*). D_17_-D_100_ are different BGRs, D_mean_ is a mean value of these BGRs. Letters (small = prop, capitals = incub) indicate the results of the Least Significant Difference t-Test. Means with the same letter are not significantly different.

The simulation of a 100-year scenario with parameters k and η resulted in a range of 0.18% w/w between the lowest and highest C_org_ concentration (Fig 3). The range between the parameters predicted with chemical BGR properties was the same.

## 4 Discussion

### 4.1 Quality of parameters based on incubation results

The decomposition of FOM was determined by its turnover coefficient k. The higher the k value, the higher the velocity of the FOM turnover process. Our estimated k values were slightly higher than the k values used for other organic materials in the CANDY process model. Regrettably, there are no references in the literature to compare with our results. An explanation could be that during anaerobic digestion, the complex organic materials, such as carbohydrates, lipids and fats, cracked into monomers and then into fatty acids followed by degradation into biogas [10], thus providing more easily decomposable compounds. However, due to model settings, the synthesis coefficient η has a stronger influence on the carbon reproduction flux than on the turnover coefficient k [18]. Thus, we focus on η in our further discussion.

The FOM decomposition results in the creation of SOM. This part of the carbon flux was mainly determined by the synthesis coefficient η. The bigger the η value, the more FOM carbon was integrated into SOM. In Sänger, Geisseler [16], the amount of emitted CO_2_ relative to the supplied carbon (up to 22.2%) was slightly lower compared to other authors [22–24]. Thus, the contribution to SOM storage was high, with correspondingly high η values. In the CANDY model, the following parameter values are integrated: η = 0.6 for cattle manure and η = 0.65 for cattle slurry [25]. Nielsen, Sensel-Gunke [26] classified the biodegradability of BGRs between cattle slurry and cattle manure. In contrast, Chen, Blagodatskaya [27] showed in a 21 days incubation experiment that in BGR-treated soils, only 6.4% of the initial carbon input was mineralized compared to 30% of the initial mineralized carbon in maize straw-treated soils. This means that BGRs had a higher η than maize straw (η of maize straw = 0.62 in CANDY), which corresponds to our findings with an η range between 0.8 and 0.89. Our findings are also confirmed by the observations of De la Fuente, Alburquerque [28], who reported that approximately 60% more carbon was mineralized in cattle slurry-treated soil than in BGR-treated soil after 56 days of incubation.

In the experiment used for this study, only the total carbon of the BGRs was measured, so no information about inorganic carbon concentration was available. Carbonates in BGR can represent up to 7.6% of dry matter depending on the substrate mix [29]. The incorporation of BGRs with a pH value of 7.5–7.8 into a relatively acid soil with a pH value of 5.5–6.5 can very likely be accompanied by the reaction of carbonate with protons to form CO_2_ and H_2_O [30]. This CO_2_ can be mistakenly interpreted as organic carbon degradation, and consequently, carbon mineralization rates could be over-estimated. This could be an error source in this study, since higher mineralization rates would mean smaller η values.

### 4.2 Quality of parameter estimation based on chemical BGR properties

Soil organic matter results from FOM turnover is a result of microbial productivity. It is composed of plant residues, microbial compounds and molecules resulting from biological degradation [31–33]. When applied to soil, microorganisms start to utilize the BGRs, which are rich in microbial biomass [34]. The efficiency of the microbial organic matter turnover depends on the quality of the FOM, the microbial community composition and environmental conditions [35].

The parameter k was related to pH. The pH value can be an important factor of environmental conditions for the microorganisms. Biogas production is performed using complex microbial communities that need different pH values for optimal performance during the different organic matter degradation stages [10]. In soil, the pH value influences a number of factors affecting microbial physiological status, microbial activity, like solubility, and the ionization of inorganic and organic solution constituents; these, in turn, affect soil enzyme activity [36, 37]. The pH influence on several processes during anaerobic digestion, as well as on the soil, are not a complete explanation, but it is still reasonable to relate the k parameter to this generally available property.

The parameter η was related to the C/N_org_ ratio. It is well known that the C/N ratio is important for microbial decomposition. Bacterial biomass generally has a much lower C/N ratio (3.5:1 to 7:1) than fungi (10:1 to 15:1), plant residues or soil [37, 38]. The stronger the processing and decomposition of the fermenter feedstock in the biogas plant, the lower the C/N ratio, the higher the NH_4_^+^ concentration and pH value, and consequently, the lower the concentration of microbial biomass in the remaining BGR [39]. The BGRs used in this study have C/N ratios between 10 and 15.6. In the model, the C/N ratio of the decomposable SOM is fixed at 8.5, which is similar to the C/N of microorganisms [18]. The average fungal carbon to bacterial carbon ratio for BGRs is 0.29 [39]. This means that microbial carbon consists of 23% fungal carbon and 77% bacterial carbon, neglecting the possible presence of Archaea [39]. Thus, when the C/N ratio is high, fungal development is favored over bacterial development [40, 41]. Therefore, increasing C/N ratios means consequently decreasing η values (Fig 2B).

**Fig 4:**
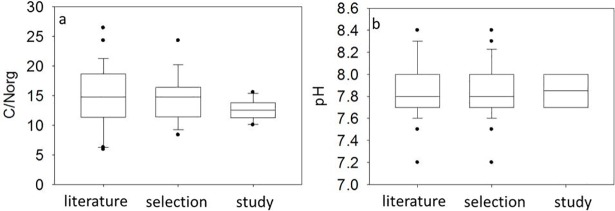
(a) C/N_org_ and (b) pH distribution of all BGRs found in the literature (literature), BGRs that are produced from the same substrates as in this study (selection) and BGRs used in our study (study) (see also S2, S3 and S4 Tables).

This study represents the first attempt to characterize BGR organic turnover parameters for process modeling. In doing so, we tried to cover the whole spectrum of animal excrement and plant-based BGRs. We compared our dataset with BGRs published in the literature (Fig 4). An examination of 85 BGRs from the literature indicate that the range and average pH values in our study are similar to those published [4, 27–29, 42–45]. In terms of C/N_org,_, we found a sample of 23 BGRs with a range between 5.9 and 26.4, which exceeds the range of BGRs used in this study [4, 28, 29,44–46]. The BGRs chosen from Sänger, Geisseler [16] cover only 27% of the C/N_org_ range found in the literature. If we take into account only BGRs that are produced from the same substrates, as in this study, and neglect BGRs derived, for example, from municipal waste, our predicted results cover 35% of the C/N_org_ values.

### 4.3 Scenario simulation

The scenario simulation showed that the BGRs used as organic fertilizer will lead to C_org_ changes in the range of 0.18% w/w after 100 years under the assumed conditions. We found significant C_org_ differences between the BGRs (Fig 3). The error bars for variants where estimations were based on chemical BGR properties were bigger than for variants where the parameters were calculated from inverse modeling. Nonetheless, these uncertainties are smaller than the model error and smaller than errors from measurement of C_org_ in the soil samples [47].

However, we covered only 27% of all BGRs for parameter η. In general, looking at the high diversity of BGRs, the resulting range of C_org_ values at the end of the scenario can be expected to be even greater. This means that the usage of one general parameter set for all BGRs may lead to considerable errors in SOM change modeling.

## 5 Conclusions

Any assessment of BGRs has to take into account the high potential variability within this substrate group. In order to predict the effect on SOM turnover, we determined the values for the turnover coefficient k and the synthesis coefficient η and found a linear relationship between those parameters and selected chemical properties of BGRs. Nevertheless, we recommend conducting incubation experiments for each BGR type as basis for the estimation of carbon turnover parameters As this is very time consuming it may be an useful approach to estimate the parameters using the proposed functions based on the chemical composition (see 5.2) instead of one general value to represent all BGRs. The preferred method should depend on the purpose and the required precision of the results. The parameter estimation from chemical composition is easily available and can be beneficial for practical applications. Both of the suggested properties can be determined by simple analytical means. Incubation experiments are time consuming, but the parameters can be defined more precisely. The results of this study are preliminary, but to the best of our knowledge, there is currently no BGR parameterization solution available. However, since our results only partially cover the variability of BGRs, more research about the carbon turnover of BGRs on lab and field scale is required to validate or improve our results.

## Supporting information

S1 Table Raw data (results) from inverse modeling for all digestates from [16]. K and η were used for means, sd and RMSE calculation (Table 3).
(XLSX)

S2 Table C/N_org_ and pH values from published literature which were used for Fig 4 (literature). (XLSX)

S3 Table C/N_org_ and pH values from digestates which were used in this study (selection). (XLSX)

S4 Table C/N_org_ and pH values from all digestates which were published in [16] (study). (XLSX)
